# Antipsychotic, antidepressant, and cognitive-impairment properties of antipsychotics: rat profile and implications for behavioral and psychological symptoms of dementia

**DOI:** 10.1007/s00210-014-0966-4

**Published:** 2014-03-06

**Authors:** Marcin Kołaczkowski, Paweł Mierzejewski, Przemyslaw Bienkowski, Anna Wesołowska, Adrian Newman-Tancredi

**Affiliations:** 1 Adamed Ltd, Pienków 149, 05-152 Czosnów, Poland; 2 Faculty of Pharmacy, Jagiellonian University Collegium Medicum, 9 Medyczna Street, 30-688 Cracow, Poland; 3 Department of Pharmacology, Institute of Psychiatry and Neurology, 9 Sobieskiego Street, 02-957 Warsaw, Poland; 4 NeuroAct Communication, 81100 Castres, France

**Keywords:** Dementia, Antipsychotic, Depression, Cognition, Behavior

## Abstract

Many dementia patients exhibit behavioral and psychological symptoms (BPSD), including psychosis and depression. Although antipsychotics are frequently prescribed off-label, they can have marked side effects. In addition, comparative preclinical studies of their effects are surprisingly scarce, and strategies for discovery of novel pharmacotherapeutics are lacking. We therefore compared eight antipsychotics in rat behavioral tests of psychosis, antidepressant-like activity, and cognitive impairment as a basis for preclinical evaluation of new drug candidates. The methods used in this study include inhibition of MK-801-induced hyperactivity, forced swim test (FST), passive avoidance (PA), spontaneous locomotor activity, and catalepsy. The drugs exhibited antipsychotic-like activity in the MK-801 test but with diverse profiles in the other models. Risperidone impaired PA performance, but with some dose separation versus its actions in the MK-801 test. In contrast, clozapine, olanzapine, lurasidone, and asenapine showed little or no dose separation in these tests. Aripiprazole did not impair PA performance but was poorly active in the MK-801 test. Diverse effects were also observed in the FST: chlorpromazine was inactive and most other drugs reduced immobility over narrow dose ranges, whereas clozapine reduced immobility over a wider dose range, overlapping with antipsychotic activity. Although the propensity of second-generation antipsychotics to produce catalepsy was lower, they all elicited pronounced sedation. Consistent with clinical data, most currently available second-generation antipsychotics induced cognitive and motor side effects with little separation from therapeutic-like doses. This study provides a uniform in vivo comparative basis on which to evaluate future early-stage drug candidates intended for potential pharmacotherapy of BPSD.

## Introduction

Dementia is a syndrome of progressive deterioration of cognitive abilities associated with psychiatric and behavioral disturbances and difficulties in carrying out daily functions (Hersch and Falzgraf 2007). In view of increased life expectancy and population aging, dementia represents a growing medical problem. Indeed, the global prevalence of dementia is estimated to be 3.9 % in individuals over the age of 60 years (Ferri et al. 2005), and a large majority of these (as many as 60 % of community-dwelling dementia patients) also experience behavioral and psychological symptoms of dementia (BPSD) at some time. The most troublesome symptoms include psychosis (delusions and hallucinations), depression, dis-inhibition, irritability, verbal and physical aggression, agitation, and anxiety (Jeste et al. 2008). Between 40 and 60 % of patients suffering from dementia experience depressive symptoms at some stage of the disease (Hersch and Falzgraf 2007), and the prevalence of psychotic symptoms can reach 63 % for delusions and up to 41 % for hallucinations in certain patient populations (Jeste et al. 2008).

Until the mid-1990s, first-generation antipsychotics were the drugs of choice for BPSD treatment, especially when delusions and hallucinations were present. Indeed, although haloperidol has no effect on agitation or behavioral symptoms as a whole, it reduces aggression. Other meta-analyses indicated no major difference between first-generation antipsychotics in efficacy for BPSD (Sink et al. 2005).

Subsequently, second-generation ‘atypical’ antipsychotics partially replaced first-generation antipsychotics in the treatment of BPSD (De Deyn et al. 2005). A Cochrane review (Ballard and Waite 2006) of 16 clinical trials with atypical antipsychotics in the treatment of BPSD revealed that risperidone and olanzapine were effective in the treatment of aggression, and risperidone was superior to placebo in the treatment of dementia-related psychosis. However, both drugs elicited significantly increased adverse cardiovascular events and extrapyramidal symptoms (EPS). In comparison, aripiprazole showed no benefits over placebo in controlling delusions and hallucinations in patients with Alzheimer’s disease and psychosis (De Deyn et al. 2005). The use of antipsychotics in BPSD is further complicated by the fact that these drugs often exacerbate preexisting cognitive deficits and produce EPS (Fasano et al. 2012; Jeste et al. 2008), emphasizing the view that currently marketed antipsychotics may not constitute acceptable therapies for elderly patients with BPSD (Gareri et al. 2014). In fact, since 2005, the US Food and Drug Administration (FDA) has required the inclusion of alerts on the package inserts of atypical antipsychotics drugs. Such “boxed warnings” are related to the risk of serious adverse effects that can be induced by atypical antipsychotics being prescribed among elderly patients with dementia. Nevertheless, antipsychotics continue to be widely prescribed for BPSD (Schneider et al. 2006b; Schulze et al. 2013b), no doubt largely due to the absence of improved drugs with safer and more effective therapeutic profiles (Schulze et al. 2013a).

Meeting the medical need for efficacious pharmacotherapeutics to treat BPSD would require the development of drug discovery strategies for evaluation of novel compounds in experimental tests relevant to the disorder. Specifically, novel drugs should alleviate psychotic and/or depressive-like symptoms and not interfere with cognitive or motor performance (Fasano et al. 2012; Jeste et al. 2008). However, drug discovery efforts in this area are in their infancy, and no recognized animal models of BPSD are currently available, complicating early first-in-vivo drug screening. Hence, pharmacological profiling of novel drug candidates must currently rely on a battery of known tests that address different symptoms. Such a strategy is described in a recent publication characterizing the profile of ADN-1184, a novel compound with potential anti-BPSD activity (Kołaczkowski et al. 2013). The compound was active in models of antipsychotic-like activity without eliciting catalepsy or impairment of passive avoidance (PA) performance. In the rat forced swim test (FST), ADN-1184 decreased immobility time, suggesting that it possesses some antidepressant-like activity. However, results on ADN-1184 need to be compared with those on existing antipsychotics characterized under uniform conditions in order to determine if the compound has distinguishing features that suggest potential therapeutic superiority. However, relevant comparative pre-clinical pharmacological data are surprisingly scarce and fragmentary, and parallel studies concerning the effects of antipsychotics in rodent models of psychosis, depression, and memory are largely absent. In addition, novel drugs such as asenapine and lurasidone (Ishibashi et al. 2010; Marston et al. 2009) have recently become available and remain to be fully characterized. Lurasidone is reputed to display favorable activity in models of cognitive activity (Yuen et al. 2012), but replication studies from other laboratories are mostly lacking. There is therefore a need to characterize established antipsychotics using the same animal models that were used to characterize ADN-1184 and that are commonly used as first-line screening procedures for novel psychotropic agents.

Here, we characterized the antipsychotic-like activity of eight antipsychotics using inhibition of hyperlocomotion induced by the non-competitive *N*-methyl-d-aspartate (NMDA) receptor antagonist, dizocilpine (MK-801) (Andiné et al. 1999). Effects of drugs on spontaneous activity and catalepsy induction were recorded in order to assess their potential to induce motor deficits and EPS. We used reduction of immobility in the FST as a measure of antidepressant efficacy and disruption of performance in the PA test as a measure of the drugs’ potential for impairing cognitive processes (Ishiyama et al. 2007; Porsolt et al. 1978; Schatzberg and Nemeroff 2009). Imipramine was used as comparator for the FST, and scopolamine was used as comparator in PA experiments.

Two first-generation antipsychotics (haloperidol, chlorpromazine) were compared with well-established second-generation atypical antipsychotics (clozapine, olanzapine, risperidone, aripiprazole) and with lurasidone and asenapine, which have been recently introduced to the market (Ishibashi et al. 2010; Schatzberg and Nemeroff 2009). As stated above, a lack of clear-cut separation between doses producing antidepressant and antipsychotic effects and doses leading to deterioration of cognitive and motor functions can limit the use of antipsychotic drugs for BPSD (Ballard et al. 2009; Jeste et al. 2008; Potenza and McDougle 1998). Therefore, a key aim of the present study was to asses the relationship between antipsychotic, antidepressant, motor impairing, and amnestic doses of a wide range of antipsychotic drugs.

## Methods

### Subjects

Drug-naive male Wistar rats (Charles River, Sulzfeld, Germany) weighing 200–225 g on arrival were used (*n* = 7–8 rats per group). Animals were supplied by the breeder 2–3 weeks before the onset of behavioral procedures. Rats were housed four per standard plastic cage and kept in a room with constant environmental conditions (22 ± 1 °C, relative humidity 60 %, a 12:12 light–dark cycle with lights on at 7:00 a.m.). During this time, the subjects were weighted and handled several times. Tap water and standard lab chow (Labofeed H, WPIK, Kcynia, Poland) were available ad libitum. All tests were carried out in a sound-attenuated experimental room between 09:00 a.m. and 3:00 p.m. Treatment of rats in the present study was in full accordance with the ethical standards laid down in respective Polish and European (Directive no. 86/609/EEC) regulations. All procedures were reviewed and approved by a local ethics committee.

### MK-801-induced hyperlocomotion

The tests used in the present study were recently described by Kołaczkowski et al. (2013). Antipsychotic-like activity was assessed by inhibition of the hyperactivity elicited by the NMDA receptor antagonist, MK-801 (Andiné et al. 1999). Briefly, groups of drug-naive rats (*n* = 7–8) were transferred in their home cages to the experimental room 24 h prior to testing and allowed to habituate for 60 min and returned to colony room. The next day, locomotor activity was assessed in black octagonal open fields (80 cm in diameter, 30 cm high) under dim light and continuous white noise (65 dB). Each animal was placed in the central part of the open field and allowed to freely explore the whole area for 30 min. Subjects did not have visual contact with other rats during the experiment. Forward locomotion (cm/30 min) was registered and analyzed with the aid of the computerized video tracking system (Videomot, TSE, Bad Homburg, Germany).

Rats were pre-administered i.p. or s.c. with antipsychotic drug or its vehicle 60 min before the start of the locomotor activity test. Fifteen minutes before the start of the test, rats were administered MK-801 (0.3 mg/kg i.p.). To assess spontaneous locomotor activity, rats were administered saline 15 min prior the test.

### Forced swimming test

The procedure used in the study to determine antidepressant-like activity was a modification of the technique described by Porsolt et al. (1978). Briefly, rats were individually placed in glass cylinders (40 cm in height, 17 cm in diameter) filled with water (temperature 23 ± 1 °C) at a height that made it impossible to reach the bottom with hind paws (25 cm). There were two swimming sessions separated by 24 h: an initial 15-min pre-test and a 5-min test. The duration of immobility (s) in the test session was recorded by a blinded observer located in an adjacent room with the aid of a video camera. A rat was considered immobile when it floated not moving except to keep the head above the water surface. Animals were injected with vehicle or drugs 60 min. i.p. or s.c. before the test.

### Step-through passive avoidance test

Effects of antipsychotics on memory function were evaluated using a step-through passive avoidance test (Ishiyama et al. 2007). Briefly, the passive avoidance apparatus (PACS-30, Columbus Instruments, Columbus, OH, USA) comprised four identical stainless-steel cages with black Plexiglas covers. Each cage consisted of a lighted and a dark compartment (23 × 23 × 23 cm) and a stainless-steel grid floor. The two compartments were separated by an automated sliding door. In the training (acquisition) session, the animals were individually placed in the lighted compartment and allowed to explore it freely for 10 s. The sliding door was then opened, and the step-through latency for animals to enter the dark compartment was measured with a 300-s cutoff time. As soon as the animals entered the dark compartment, the door was closed. An inescapable foot-shock (0.5 mA pulse monopolar current for 3 s) was delivered 3 s later through the grid floor with a monopolar current shock generator (EACS-30, Columbus Instruments). The tested compound, or its vehicle, was administered 60 min before the start of the training session. All control vehicle-treated animals entered the dark compartment during the training session and received a foot-shock. Drug-treated animals that did not enter the dark compartment in the training session were not subjected to the test session. In the present study, all the tested animals entered the dark side, except those that had been treated with haloperidol at doses of 0.3 and 1.0 mg/kg. At these doses, 50 % of the rats entered the dark compartment.

The test session was performed 24 h after the training session using the same paradigm but without the foot-shock and drug/vehicle injections (Ishiyama et al. 2007). Step-through latencies for animals to enter the dark compartment were measured with a 300-s cutoff time. Drug-induced decreases in step-through latencies to enter the dark compartment in the test session were treated as a measure of drug’s “amnestic” effects.

### Catalepsy

Cataleptogenic responses were assessed using the bar test. Each rat was placed on a clean, smooth table with the wooden bar (2 × 3 × 25 cm, *H* × *W* × *L*) suspended 10 cm above the working surface. The animal’s hindlimbs were freely placed on the table, the tail laid out to the back, and the forelimbs gently placed over the bar. The length of time the animal touched the bar with both front paws was measured up to a cutoff time of 180 s. Results of each trial were scored as follows: 0 for holding the position for <15 s, 1 for holding it for 15–29.9 s, 2 for holding it for 30–59.9 s, and a maximum score of 3 for staying on the bar for >60 s (Ogren et al. 1986). The minimum cataleptogenic dose was defined as the lowest dose inducing a mean catalepsy score of ≥1. Catalepsy was scored 30, 60, and 120 min after administration of vehicle or a test drug.

### Data analysis

Data were analyzed by one-way analysis of variance (ANOVA). The Newman–Keuls test was used for post hoc comparisons. Induction of hyperlocomotion by MK-801 (MK-801 vs. saline) was confirmed by Student’s *t* test. The reversal of MK-801-induced hyperactivity by antipsychotic drugs was analyzed by ANOVA. In the case of the passive avoidance test, data were not normally distributed so step-through latencies were analyzed with the aid of the Kruskal–Wallis and Mann–Whitney non-parametric tests. *p* values ≤0.05 were considered significant. The Statistica 8.0 software package for Windows (StatSoft, Tulsa, OK, USA) was used to analyze all data. The lowest drug dose eliciting a significant effect was defined as a minimal effective dose (MED).

### Drugs

MK-801 (Sigma-Aldrich, Poznan, Poland) was dissolved in sterile physiological saline (0.9 % NaCl; Baxter, Warsaw, Poland) and administered i.p. in a volume of 1.0 ml/kg. Antipsychotic drugs were also administered i.p. (except haloperidol s.c.) in injection volumes that were adjusted to the minimum necessary to ensure full solution of the compounds in the vehicle. Unless stated, all drugs were synthesized by Adamed Ltd. Aripiprazole, olanzapine, risperidone, lurasidone, and asenapine were suspended in a 1.5 % aqueous solution of Tween 80 (Sigma-Aldrich) and administered in a volume of 1 ml/kg (olanzapine, risperidone) or 2 ml/kg (aripiprazole, lurasidone, asenapine). Clozapine, was suspended in a 3 % aqueous solution of Tween 80 with a few drops of glacial acetic acid and administered i.p. in a volume of 3 ml/kg. Chlorpromazine (ampoules 25 mg/ml; Fenactil, WZF Polfa S.A., Warsaw, Poland) was diluted with physiological saline and administered i.p. in a volume of 1.2 ml/kg. Haloperidol (ampoules 5 mg/ml; Haloperidol WZF, WZF Polfa S.A.) was diluted with physiological saline and administered in a volume of 1 ml/kg. Imipramine hydrochloride and (−)-scopolamine hydrobromide trihydrate (Sigma-Aldrich) were dissolved in physiological saline and administered i.p. in a volume of 2.0 ml/kg (control animals received vehicle in the same volume as drug-treated animals). All doses refer to the quantity of free base except for chlorpromazine and lurasidone (hydrochloride salts, as clinically used).

## Results

### Antipsychotic-like activity: MK-801-induced hyperlocomotion

**Fig. 1:**
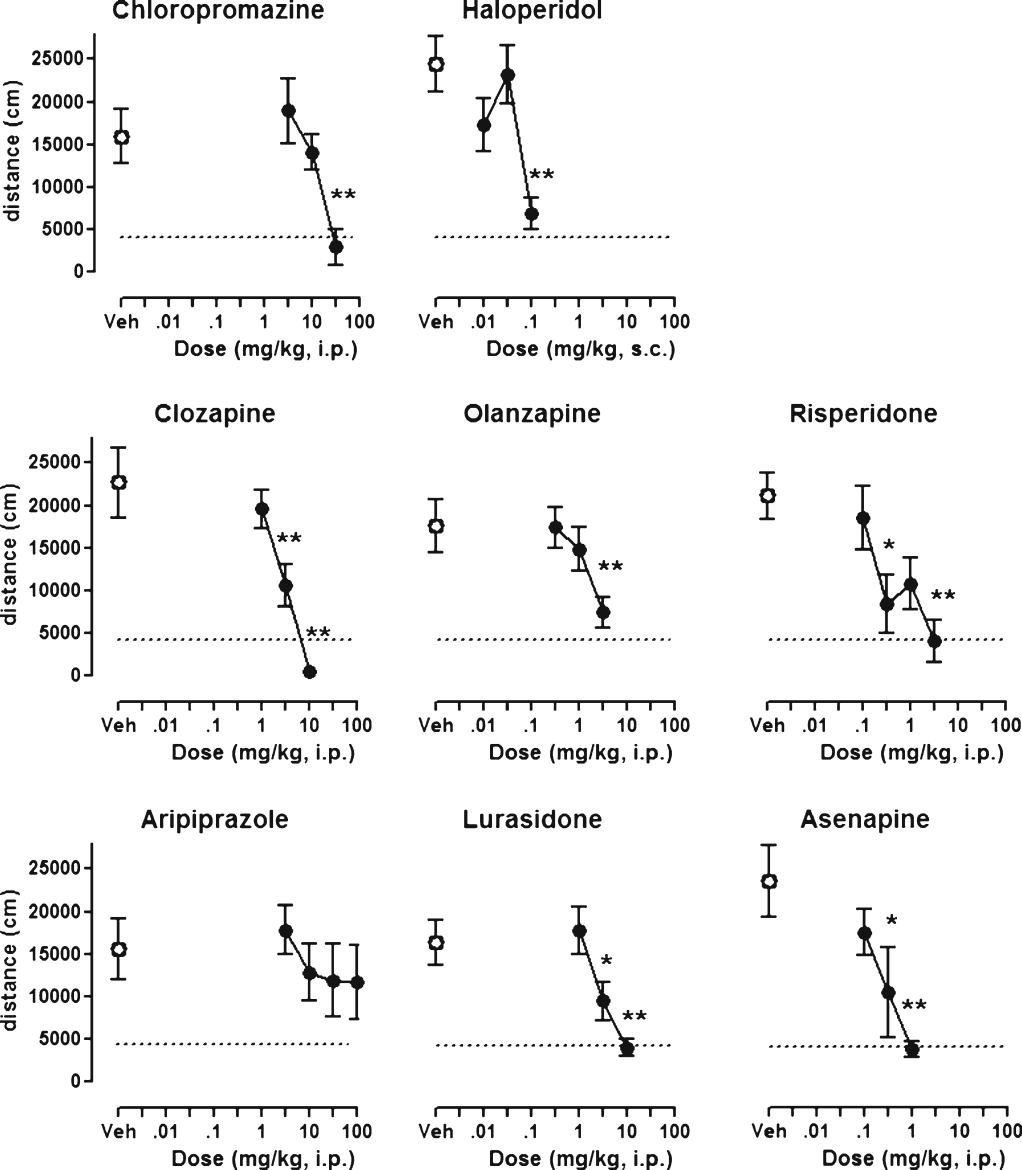
Effects of antipsychotics on hyperlocomotion induced by MK-801. Each *symbol* represents mean ± SEM distance traveled (*n* = 7–8) in the 16–45-min period after MK-801 administration. Test compound or vehicle was administered 45 min before MK-801 (0.3 mg/kg). *p < 0.05, **p < 0.01, compared with MK-801-injected control group using Newman–Keuls post hoc test, following significant ANOVA. The *lower dotted lines* represent the average locomotor activity of vehicle/saline groups

As expected (Schatzberg and Nemeroff 2009), MK-801 dose-dependently increased forward locomotion activity in all the tested groups. MK-801-treated animals showed a significant increase in distance travelled (Student’s *t* test, all *T*’s > 2.6, *p*’s < 0.05). All drugs, except aripiprazole, dose-dependently and significantly (all *F*’s > 3.6, all *p*’s < 0.05) antagonized hyperlocomotion induced by MK-801 (Fig. 1; Table 2). Haloperidol, risperidone, and asenapine were the most potent in antagonizing MK-801-induced hyperlocomotion, and chlorpromazine was the least potent (Table 1). Aripiprazole did not significantly diminish MK-801-induced hyperlocomotion even at a high dose (100 mg/kg i.p.) [*F*(4, 35) = 0.51, *p* > 0.05].

**Table 1:** Action of antipsychotic drugs on five behavioral tests in rat

	MK-801 hyperlocomotion	Spont. loco.	Catalepsy	Forced swim test		Passive avoidance	MED ratio PA/MK
	MED	MED	MED	FST: active doses	Immobility: % of vehicle (% of imipramine)	MED	
Chlorpromazine	30.0 (3–30)	3.0 (1–10)	30 (3–30)	Not active (1–10)	Not active	30.0 (3–30)	1
Haloperidol	0.1 (0.01–0.1)	0.03 (0.01–0.1)	0.1 (0.03–0.3)	0.003, 0.01 (0.003–0.1)	−7 ± 2 (28)	0.3 (0.1–1)	3
Clozapine	3.0 (1–10)	10 (1–10)	>100^a^ (10–100)	10.0, 30.0 (0.3–30)	−11 ± 5 (44)	10 (1.0–10)	3
Olanzapine	3.0 (0.3–3)	1 (0.3–3)	10 (1–10)	1.0 (0.3–3)	−11 ± 4 (44)	3.0 (1–10)	1
Risperidone	0.3 (0.1–3)	1 (0.3–3)	10 (0.3–10)	0.3 (0.1–3)	−12 ± 6 (48)	3 (0.3–3)	10
Aripiprazole	>100 (3–100)	10 (3–100)	>100 (10–100)	3.0 (1–30)	−14 ± 5 (56)	>100 (10–100)	n.d.
Lurasidone	3.0 (1–10)	3.0 (1–10)	>100 (10–100)	1.0 (0.3–3)	−14 ± 3 (56)	10.0 (1–10)	3
Asenapine	0.3 (0.1–1)	1.0 (0.1–1)	3 (1–10)	0.1 (0.03–0.3)	−12 ± 4 (48)	0.3 (0.1–1)	1
Imipramine	–	–	–	10 (3–10)	−25 ± 8 (100)	–	–
Scopolamine	–	–	–	–	–	0.3 (03–3)	–

Numbers are minimal effective doses (MED) in milligrams per kilogram. Unless otherwise indicated, numbers in brackets refer to the dose range tested expressed as milligrams per kilogram. The MED ratio column shows that for most drugs, there is little separation between doses that elicit antipsychotic-like activity in the MK-801 test and doses that impair memory performance in the passive avoidance (PA) test. Imipramine ad scopolamine are shown as comparators for the FST and PA tests, respectively

*Spont. Loco* spontaneous locomotion, *n.d.* not determined

^a^Seizures were noted in some rats

### Antidepressant-like activity: forced swimming test

**Fig. 2:**
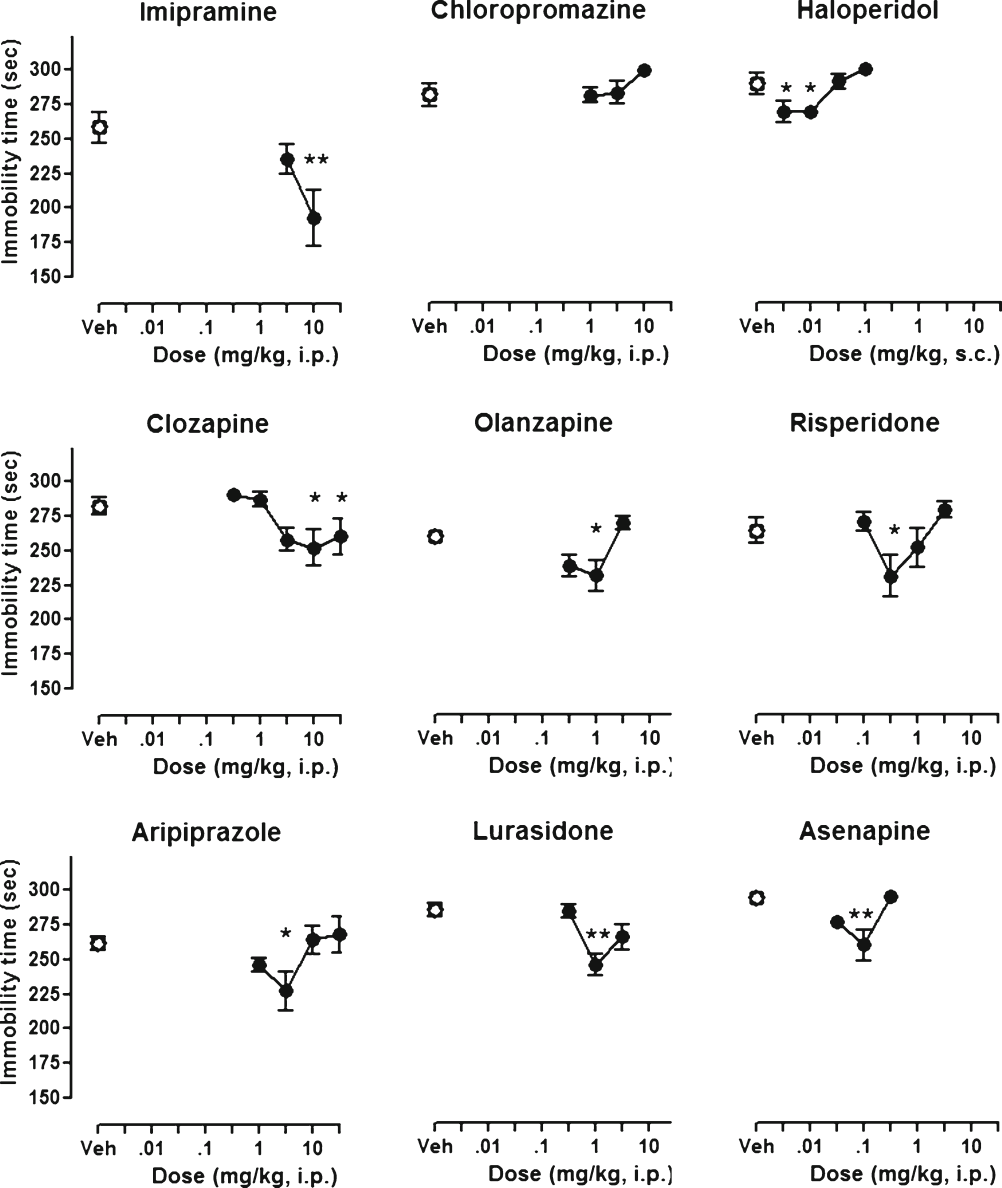
Effects of imipramine and antipsychotics on forced swimming test. Each *symbol* represents mean ± SEM immobility time during 5-min forced swimming session (*n* = 7–8). Imipramine was administered 30 min before the test, whereas the other compounds were administered 1 h before the test. *p < 0.05, **p < 0.01, compared with vehicle-injected control group using Newman–Keuls post hoc test, following significant ANOVA

Duration of immobility in vehicle-treated subjects was 258–292 s. The tricyclic antidepressant, imipramine, dose-dependently reduced immobility in the FST, consistent with antidepressant-like properties (Porsolt et al. 1978). Maximal reduction of immobility by imipramine at 10 mg/kg was 25 % of control values (Table 2; Fig. 2) (*F*(2, 21) = 5.1, *p* < 0.05, the post hoc Newman–Keuls test revealed significant effect at dose 10 mg/kg, *p* < 0.01). Among the antipsychotic drugs, chlorpromazine did not reduce the duration of immobility in this test (*F*(3, 28) = 1.8, *p* > 0.05). All other compounds modestly, but significantly, reduced immobility time at least at one dose (all *F*’s > 3.2, *p*’s < 0.05). Notably, most of the tested antipsychotics evoked reduction in immobility time with U-shaped dose–response curves, as shown in Fig. 2. In contrast, clozapine evoked significant antidepressant-like effects at the two highest doses tested, 10 and 30 mg/kg.

**Table 2:** Statistical summary of behavioral effects of drugs

Drug	MK-801 effect of MK-801 vs. saline	MK-801: effect of antipsychotics vs. vehicle	Spontaneous locomotor activity	Forced swim test	Step-through latency training session	Step-through latency test session
Chloropromazine	*t*(14) = 3.2, *p* < 0.01	*F*(3, 28) = 5.9, *p* < 0.01	*F*(3, 28) = 12.66, *p* < 0.01	*F*(3, 28) = 1.8, *p* > 0.05	*H*(3) = 8.96, *p* < 0.05	*H*(3) = 12.6, *p* < 0.01
Haloperidol	*t*(14) = 2.6, *p* < 0.05	*F*(3, 28) = 13.7, *p* < 0.01	*F*(3, 28) = 31.80, *p* < 0.01	*F*(4, 35) = 7.2, *p* < 0.01	*H*(3) = 18.2, *p* < 0.01	*H*(3) = 17.7, *p* < 0.01
Clozapine	*t*(14) = 4.0, *p* < 0.01	*F*(3, 28) = 13.8, *p* < 0.01	*F*(3, 28) = 5.50, *p* < 0.01	*F*(5, 42) = 4.2, *p* < 0.01	*H*(3) = 1.3, *p* > 0.05	*H*(3) = 10.7, *p* < 0.05
Olanzapine	*t*(14) = 4.2, *p* < 0.01	*F*(3, 28) = 3.6, *p* < 0.05	*F*(3, 28) = 6.10, *p* < 0.01	*F*(3, 28) = 5.2, *p* < 0.01	*H*(3) = 5.7, *p* > 0.05	*H*(3) = 7.1, *p* > 0.05
Risperidone	*t*(14) = 6.3, *p* < 0.01	*F*(4, 35) = 5.1, *p* < 0.01	*F*(3, 26) = 7.50, *p* < 0.01	*F*(4, 35) = 3.1, *p* < 0.05	*H*(3) = 11.5, *p* < 0.05	*H*(3) = 11.2, *p* < 0.05
Aripiprazole	*t*(14) = 4.8, *p* < 0.01	*F*(4, 35) = 0.5, *p* > 0.05	*F*(4, 39) = 12.10, *p* < 0.01	*F*(4, 34) = 2.9, *p* < 0.05	*H*(3) = 9.6, *p* < 0.05	*H*(3) = 3.0, *p* > 0.05
Lurasidone	*t*(14) = 3.2, *p* < 0.01	*F*(3, 28) = 7.7, *p* < 0.01	*F*(3, 28) = 10.8, *p* < 0.01	*F*(3, 28) = 6.5, *p* < 0.01	(3) = 3.0, *p* > 0.05	*H*(3) = 10.0, *p* < 0.05
Asenapine	*t*(14) = 4.6, *p* < 0.01	*F*(3, 28) = 5.6, *p* < 0.01	*F*(3, 28) = 8.1, *p* < 0.01	*F*(3, 28) = 6.5, *p* < 0.01	*H*(3) = 9.3, *p* < 0.05	*H*(3) = 13.3, *p* < 0.05
Imipramine	n.t.	n.t.	n.t.	*F*(2, 21) = 5.1, *p* < 0.05	n.t.	n.t.
Scopolamine	n.t.	n.t.	n.t.	n.t.	*H*(3) = 2.7, *p* > 0.05	*H*(3) = 16.5, *p* < 0.01

*n.t.* not tested

### Memory impairment: passive avoidance test

The muscarinic receptor antagonist, scopolamine, dose-dependently abolished passive avoidance response, consistent with memory impairment in this procedure (*H*(3) = 16.5, *p* < 0.01, Mann–Whitney *U* test revealed significant effects at doses 0.3 and 1.0 mg/kg, *p* < 0.05, and at dose 3.0 mg/kg, *p* < 0.01). Some antipsychotics (chlorpromazine, haloperidol, risperidone, aripiprazole, and asenapine) produced dose-dependent prolongation in step-through latency during the training (acquisition) session, suggesting motor interference consecutive to sedation or catalepsy.

**Fig. 3:**
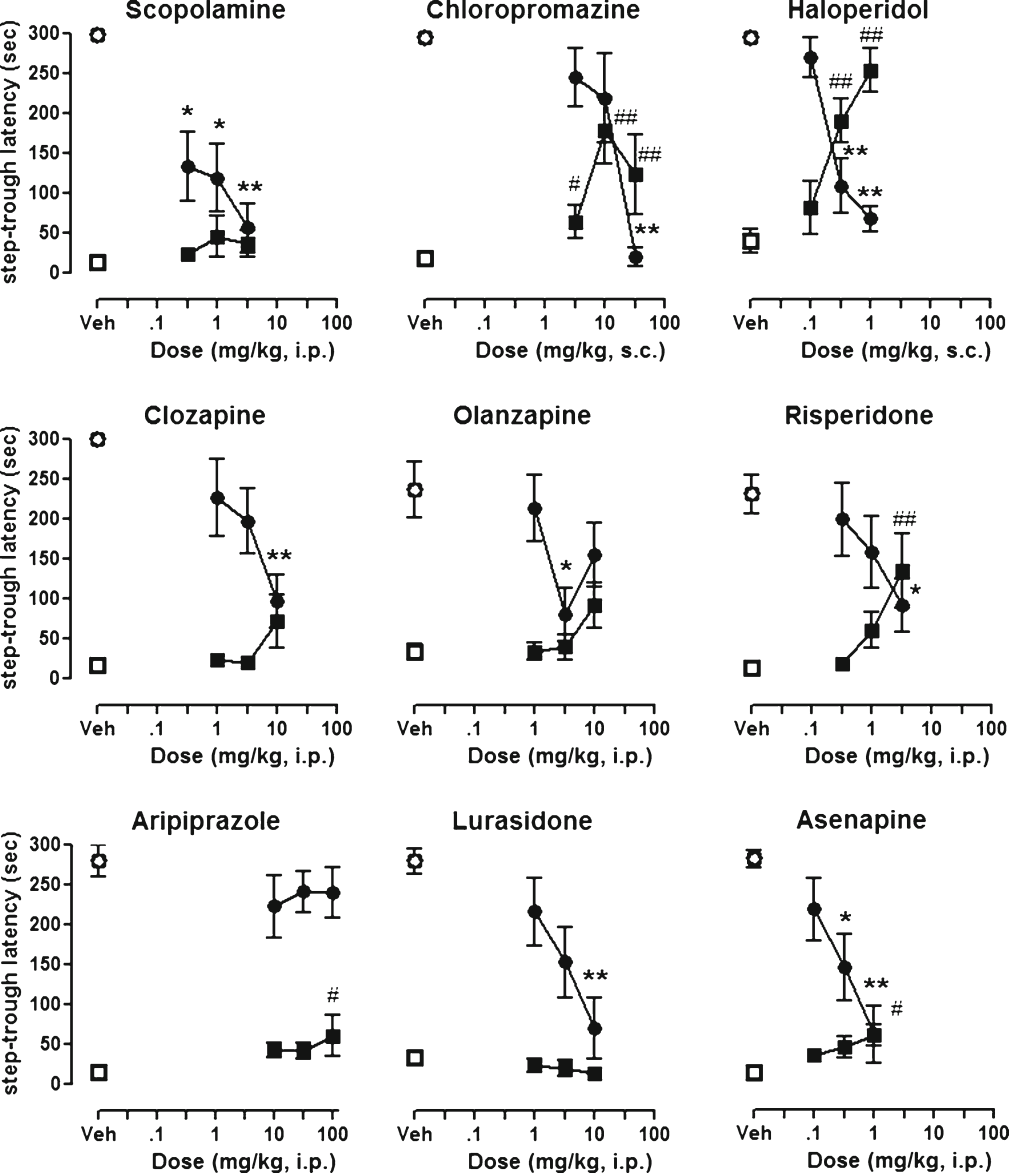
Effects of scopolamine and antipsychotics on step-through latency. Animals received antipsychotic drugs, 1 h before training session. Each *symbol* represents mean ± SEM latency to enter the dark compartment (*n* = 7–8). *Squares* indicate latency in training session, *circles* indicate latency in test sessions. *p < 0.05, **p < 0.01, compared with vehicle-injected control group using Mann–Whitney *U* test, following significant Kruskal–Wallis ANOVA

In the test (expression) sessions, all drugs, except aripiprazole, significantly (Kruskal–Wallis ANOVA: all *H*’s > 10.0, all *p*’s < 0.05) reduced the step-through latency (Fig. 3). Aripiprazole at 10–100 mg/kg did not modify the passive avoidance response (Kruskal–Wallis ANOVA *H*(3) = 3.0, *p* > 0.05). As shown in Table 1, haloperidol, risperidone, and asenapine were the most potent in reducing step-through latency.

### Movement impairment: inhibition of spontaneous locomotion and catalepsy

As expected, all antipsychotics inhibited spontaneous locomotion. In most cases, the MEDs were broadly similar to those that reduced MK-801-induced hyperactivity (Table 1). However, chlorpromazine and aripiprazole inhibited spontaneous locomotion at doses at least 10-fold lower than those active in the MK-801 test.

Most of the antipsychotics also elicited catalepsy, whereas vehicle-treated animals did not exhibit any (data not shown). The MED values were, in most cases, similar or slightly higher than those active in the MK-801 test. In contrast, clozapine, aripiprazole, and lurasidone did not elicit catalepsy even at the highest dose tested (100 mg/kg; Table 1). This dose of clozapine did, however, elicit seizures in some animals.

## Discussion

The principal finding of the present study is that currently-marketed second-generation antipsychotics offer limited separation between doses active in rat models of antipsychotic-like activity and memory impairment. Although, in accordance with clinical data, their propensity to produce catalepsy was lower, the drugs all elicited pronounced sedation. Indeed, although antipsychotics are commonly used to treat BPSD off-label, little information is available that directly compares antipsychotics in standardized pharmacological tests that may be relevant to this disorder. Thus, whereas previous studies have reported data on antipsychotic-like properties (e.g., inhibition of MK-801-induced hyperlocomotion), comparisons of these activities with those observed in PA and FST have not been thoroughly characterized. Such early-stage comparative information is important to provide a basis on which to select future drug candidates at a preclinical level. Indeed, although BPSD is a serious and unmet medical need, there is currently no consensus for identifying novel drugs with improved therapeutic potential. The present study therefore provides a comparative dataset for novel drug candidates such as the recently reported compound, ADN-1184 (Kołaczkowski et al. 2013).

### Activity of compounds in tests of antipsychotic-like efficacy and motor control

Inhibition of MK-801-induced hyperactivity was used here as a measure of antipsychotic-like activity. This test was chosen because NMDA receptors are involved in various forms of dementia (Olivares et al. 2012) and NMDA receptor density is decreased in the frontal cortex of postmortem brain samples of Alzheimer’s disease patients (Scheuer et al. 1996). This suggests that dysfunction of NMDA receptors may underlie some aspects of the disease, such as susceptibility to psychotic symptoms. In the present experiments, most of the antipsychotics abolished MK-801-induced hyperactivity, as expected. Among the recent drugs, lurasidone and asenapine, but not aripiprazole, displayed robust activity in this test. In contrast, other authors have reported significant inhibition by aripiprazole of MK-801 and ketamine-induced hyperactivity in rat and mouse (Leite et al. 2008; Nordquist et al. 2008). One reason for the discrepancy may be the somewhat variable response observed with aripiprazole herein (Fig. 1). However, the D2 receptor partial agonist properties of aripiprazole likely also render it less adapted to antagonizing hyperlocomotion elicited by NMDA receptor antagonists, as suggested by previous studies (Bardin et al. 2007; Leite et al. 2008). Indeed, the present data correlate with the modest efficacy of aripiprazole in controlling psychotic symptoms in Alzheimer’s disease patients (De Deyn et al. 2005; Schneider et al. 2006a), suggesting that reversal of NMDA receptor antagonist-induced hyperactivity is an important selection criterion for future drugs aimed at treatment of BPSD. Overall, the results from the MK-801 test are consistent with antipsychotic-like activity of the drugs and are important in the present study because they identify active doses that constitute measures of central activity for comparison with the other tests relevant to BPSD (see below).

In tests of catalepsy and inhibition of locomotor activity, the drugs displayed expected profiles: Most of the drugs elicited dose-dependent catalepsy, a model of EPS, but clozapine, aripiprazole, and lurasidone did not (Table 1). However, all the drugs tested herein inhibited spontaneous locomotor activity, probably reflecting sedative properties, at doses similar to those active in the MK-801 test (although antipsychotic-induced sedation can progressively attenuate with repeated administration). This is an issue that may be significant in the context of BPSD drug discovery because elderly patients may suffer from poor motor coordination and/or movement disorders (Fasano et al. 2012). Care is necessary when using drugs that are known to induce motor disruption, and it would therefore be desirable to identify new drug candidates that did not suffer from such motor disruption liability.

### Activity of compounds in a test of antidepressant-like efficacy

Mood deficits and depressive symptoms are very common in patients suffering from dementia (Hersch and Falzgraf 2007), and the present study shows that antipsychotics used in the treatment of BPSD have distinct effects in the FST, a classic model of antidepressant-like activity (Fig. 2). *Firstly*, the capacity of antipsychotic drugs to alleviate symptoms of depression is being increasingly recognized with the clinical use of olanzapine, risperidone, and aripiprazole to treat treatment-resistant and bipolar depression in conjunction with established antidepressants (Komossa et al. 2010) or, in the case of quetiapine, as monotherapy (Weisler et al. 2012). Clearly, inhibition of monoamine reuptake by SSRIs, SNRIs, or tricyclic drugs does not provide optimal antidepressant therapy in many patients. Indeed, established antidepressants induce a broad increase in monoamine transmission that affects all serotonergic and noradrenergic receptors, including those that limit antidepressant response, such as, for example, 5-HT6 and 5-HT7. Thus, the efficacy of adjunct treatment with antipsychotics may be due to antagonism of targets that limit antidepressant action (Carr et al. 2011).

*Secondly*, in most cases, the effects of the drugs herein were observed at only a single dose. This is likely a reflection of the multireceptor profiles of antipsychotics that can, presumably, interfere with the drugs’ capacity to alleviate mood deficits over a broad dose range. Olanzapine and lurasidone were active at doses that are 3-fold lower than those active in the MK-801 test (Table 1). In contrast, risperidone was active in the FST at the same dose that was first active in the MK-801 test. Interestingly, very low doses of haloperidol (10–30-fold lower than antipsychotic-like doses) modestly reduced immobility, probably by antagonism of pre-synaptic D2 receptors and facilitation of dopaminergic neurotransmission in limbic regions (Lucas and Spampinato 2000).

*Thirdly*, the only drug that showed significant activity at more than one dose, overlapping with antipsychotic activity, was clozapine. This antipsychotic is among the most effective in reducing suicidality, a parameter which is strongly connected to depressed mood (Meltzer 2012). Previous studies of clozapine in the FST have shown that it is also active under other experimental conditions (Chindo et al. 2012) and, in a comparative study in mice, was the only antipsychotic that showed activity in the tail-suspension test (Wesolowska et al. 2011). It therefore seems that clozapine’s mood-modulating effects are clearly measurable in pharmacological models, and this might provide a criterion by which to evaluate novel drugs at an early stage of drug discovery.

*Fourthly*, the antipsychotics reduced immobility times by about 10–15 % compared with vehicle-treated subjects. In comparison, the tricyclic antidepressant imipramine decreased immobility times by about 25 % at the dose tested. Imipramine’s effect is therefore only about 2-fold greater than that elicited by antipsychotic drugs, suggesting that the latter’s effects on mood modulation may be pharmacologically relevant. Nevertheless, higher doses of imipramine are sometimes reported to elicit larger responses so the antidepressant-like effects observed with the antipsychotics should be interpreted with caution (Kitamura et al. 2008). However, it would be interesting to evaluate the activity of clozapine and other antipsychotics in the FST upon chronic treatment—their efficacy may increase with repeated administration, as is the case for reuptake inhibitors, including imipramine (Koek et al. 1998). In addition, in view of the fact that antipsychotics are often prescribed as adjunct treatment, it would be interesting to test them in combination with clinically used antidepressants.

### Activity of compounds in a test of cognition/memory

Cognitive disturbance is a major characteristic of dementia (Hersch and Falzgraf 2007). Hence, it would be desirable to avoid treating patients suffering from BPSD with drugs that elicit or accentuate cognitive impairment. Herein, we used a classic memory test to compare the activity of the antipsychotics. The PA is based on the acquisition, storage, and retention of aversive Pavlovian conditioning involving short- and long-term memory processes. In addition, PA also depends on attention, perception (of painful stimuli and visual discrimination between the compartments), and sensorimotor integration that involves multiple neurotransmitter systems (Myhrer 2003). Therefore, PA acquisition is a composite read-out, and gaining information at an early stage on whether a compound impairs PA is of value in selection of new drug candidates for BPSD.

*Firstly*, the antipsychotics generally showed impairment of the PA response (Fig. 3; Table 1) although their pattern varied from one drug to another. Thus, risperidone impaired PA performance but did so at doses that were 10-fold greater than those active in the MK-801 test. This suggests that it can achieve antipsychotic-like activity in the absence of memory impairment, although its induction of catalepsy and spontaneous locomotor activity remain sub-optimal. Other antipsychotics, including clozapine, olanzapine, lurasidone, and asenapine, showed little (3-fold) or no MED separation between the PA and MK-801 tests. Accordingly, it would be desirable to identify drug candidates that did not disrupt memory performance in early screening tests. Such drugs could then be further characterized in diverse models of cognition relevant to dementia (see below). Also, chronic studies are warranted to assess if repeated administration affects the separation factor between doses that elicit “antipsychotic” effects and those that alter cognitive capacities.

*Secondly*, the novel antipsychotic lurasidone also impaired PA performance (MED 10 mg/kp, i.p.). This is notable because lurasidone has been claimed to exhibit a favorable cognitive profile in tests of passive avoidance, the radial arm maze, and the Morris water maze, without impairing basal PA performance (Enomoto et al. 2008; Ishiyama et al. 2007). In addition, lurasidone has been reported to attenuate MK-801-induced cognitive deficits at low doses (1–3 mg/kg p.o.) (Ishiyama et al. 2007), suggesting that it may exhibit dose separation between some of its pro-cognitive and antipsychotic-like properties. It will therefore be interesting to determine the extent to which lurasidone is of utility in dementia patients.

*Thirdly*, the striking absence of memory impairment by aripiprazole is consistent with its generally benign tolerance profile. The partial agonist properties of aripiprazole at D2 and 5-HT1A receptors are claimed to provide “stabilizing” influence on neurotransmission, and this may underlie the drug’s absence of interference on PA performance (Tamminga and Carlsson 2002). Nevertheless, the incomplete blockade of D2 receptors by aripiprazole (due to its partial agonist activity at these sites) may also render it less incisive for control of psychotic symptoms or agitated states, as reflected in its somewhat lesser capacity to reverse MK-801-induced hyperactivity, as noted above, and its lack of clinical antipsychotic efficacy in a trial of Alzheimer disease patients (De Deyn et al. 2005).

*Fourthly*, the present study examined the effects of antipsychotics on the PA test in normal rats that were drug-naïve and normally express a high level of performance. Therefore, the present conditions detect impairments of memory performance rather than cognitive enhancement (Marston et al. 2009). Ideally, drugs would be active in MK-801-induced hyperactivity tests while reducing immobility in the FST and being free of interference on (or even improving) PA performance. Such a screening battery could, theoretically, identify promising compounds for subsequent characterization in more advanced tests of anxio-depressive states and cognition. For example, drugs could then be tested for their capacity to reverse memory deficits elicited by muscarinic receptor antagonists such as scopolamine (Gravius et al. 2011) and/or examine their effects in aged animals that suffer from impairment arising from decline in cerebral function or in mice that have been genetically modified to alter beta-amyloid expression and neurologically mimic Alzheimer’s disease (Lithner et al. 2011).

### Mechanistic aspects

All of the drugs examined herein share the common property of interacting with dopamine D2 receptors, the primary action responsible for their antipsychotic-like profiles. In contrast, the drugs exhibit markedly diverse profiles of action at serotonergic receptor subtypes. In particular, the interaction of some atypical antipsychotics with 5-HT1A, 5-HT6, and 5-HT7 receptors is likely to underlie their effects on antidepressant-like behavior and cognitive performance (Arnt et al. 2008). Thus, 5-HT1A receptor activation mediates the capacity of aripiprazole to reverse social interaction deficits elicited by PCP (Newman-Tancredi and Kleven 2011) and may also underlie some of clozapine’s and lurasidone’s activity (Horiguchi and Meltzer 2012; Ishibashi et al. 2010). Lurasidone also has pronounced 5-HT7 interaction, a property which has been claimed to contribute to its attenuation of PCP-induced novel object recognition deficits (Horiguchi et al. 2011; Ishibashi et al. 2010).

Lastly, 5-HT6 antagonism is a prominent feature of the receptor profile of clozapine and has been suggested to mediate some of its “atypical” properties (Glatt et al. 1995). Indeed, 5-HT6 receptor antagonists are active in models of cognition relevant to psychotic disorders (Arnt and Olsen 2011; Rodefer et al. 2008) as well as in tests of antidepressant-like and anxiolytic activity (Carr et al. 2011). 5-HT6 receptor antagonism may also be a promising target for therapy of Alzheimer’s disease (Gravius et al. 2011): A selective 5-HT6 antagonist, LuAE58054, improved cognitive performance of Alzheimer disease patients (H. Lundbeck A/S 2012). At a drug discovery level, the novel compound, ADN-1184, possesses a promising profile in rat models (active in the MK-801 and FST tests without catalepsy or impairment of PA) and is characterized by potent antagonism of 5-HT6 and 5-HT7 receptors, suggesting that they mediate some of its therapeutic-like properties (Kołaczkowski et al. 2013).

### Conclusions and perspectives

To summarize, all the antipsychotics possess sub-optimal profiles, mostly by eliciting memory-impairment or sedation at antipsychotic-like range of doses—side effects that may be of particular concern for elderly dementia patients with preexisting cognitive deficits and motor difficulties. Improved management of BPSD may be achieved by use of drugs that incisively oppose glutamate-related psychosis but do not elicit cognitive deficits or motor impairment. If such drugs also possessed accentuated antidepressant properties, they could constitute promising therapeutics for treatment of dementia-related disturbances in elderly patients. While further investigation would be desirable to determine the effects of drugs under other treatment conditions (e.g., following repeated administration or in conjunction with antidepressants), the present comparative study provides a reference dataset by which future drugs candidates can be evaluated at an early stage of in vivo testing.

Concerning longer-term perspectives, the regulatory framework for development of new BPSD drugs requires clarification. Indeed, this should be addressed in a multidisciplinary context involving experts from pharmaceutical, medical, and social sciences. A critical point is balancing somatic risks induced by use of antipsychotics and their potential to improve clinical status and quality of life of dementia patients and caregivers. Recently, pimavanserin, a drug targeting psychosis in Parkinson’s disease patients, was granted an expedited path to NDA filing, suggesting that the regulatory environment concerning psychotropic drugs for the elderly may be changing. If so, there may be a rise in much-needed drug discovery and development of novel treatments, offering hope for the patients and families of those suffering from disorders (such as BPSD) that are associated with dementia.
